# Supplementary material for: Effect of physical activity on the risk of frailty: A systematic review and meta-analysis
Source: PLoS One. 2022 Dec 1;17(12):e0278226. doi: 10.1371/journal.pone.0278226 (PMC9714708; doi:10.1371/journal.pone.0278226)
Supplement: S1 Table — (DOCX) [file pone.0278226.s001.docx]

**Table S1. Search strategy of the study**

|  | **Pubmed/ Embase/ Web of Science search** | **Search** |
| --- | --- | --- |
| 1 | Physical activity | Title/Abstract |
| 2 | Exercise | Title/Abstract |
| 3 | Exercises | Title/Abstract |
| 4 | Training | Title/Abstract |
| 5 | Physical exercise | Title/Abstract |
| 6 | Acute exercise | Title/Abstract |
| 7 | Isometric exercises | Title/Abstract |
| 8 | Exercise training | Title/Abstract |
| 9 | Aerobic exercise | Title/Abstract |
| 10 | Combined training | Title/Abstract |
| 11 | Combined exercise | Title/Abstract |
| 12 | Weight-lifting | Title/Abstract |
| 13 | Sports | Title/Abstract |
| 14 | Running | Title/Abstract |
| 15 | Jogging | Title/Abstract |
| 16 | Swimming | Title/Abstract |
| 17 | Walking | Title/Abstract |
| 18 | Yoga | Title/Abstract |
| 19 | Tai chi | Title/Abstract |
| 20 | Endurance exercise | Title/Abstract |
| 21 | Resistance exercise | Title/Abstract |
| 22 | Strength exercises | Title/Abstract |
| 23 | Daily activity | Title/Abstract |
| 24 | Lifestyle | Title/Abstract |
| 25 | Frailty | Title/Abstract |
| 26 | Frailties | Title/Abstract |
| 27 | Frailness | Title/Abstract |
| 28 | Frailty syndrome | Title/Abstract |
| 29 | Debility | Title/Abstract |
| 30 | Debilities | Title/Abstract |
| 31 | 1 OR 2 OR 3 OR 4 OR 5 OR 6 OR 7 OR 8 OR 9 OR10 OR 11 OR 12 OR 13 OR 14 OR 15 OR 16 OR 17 OR 18 OR 19 OR 20 OR 21 OR 22 OR 23 OR 24 | |
| 32 | 25 OR 26 OR 27 OR 28 OR 29 OR 30 | |
| 33 | 31 AND 32 | |
|  | Database Searched:  Pubmed (https://pubmed.ncbi.nlm.nih.gov/)  Embase (https://www.embase.com/)  Web of Science (http://apps.webofknowledge.com/) | |
