# Supplementary material for: Effect of physical activity on the risk of frailty: A systematic review and meta-analysis
Source: PLoS One. 2022 Dec 1;17(12):e0278226. doi: 10.1371/journal.pone.0278226 (PMC9714708; doi:10.1371/journal.pone.0278226)
Supplement: S2 Table — (DOCX) [file pone.0278226.s002.docx]

**Table S2. Detailed quality assessment of the included studies by using criteria of the Newcastle-Ottawa Scale**

| Study, year | Selection | | | |  | Comparability | |  | Outcome | | | Scores |
| --- | --- | --- | --- | --- | --- | --- | --- | --- | --- | --- | --- | --- |
|  | Representativeness of the exposed cohort | Selection of the non- exposed cohort | Ascertainment of exposure | Demonstration that outcome of interest was not present at start of study |  | Select the most important factor | Study controls for any additional factor |  | Assessment of outcome | Was follow-up long enough for outcomes to occur | Adequacy of follow up of cohorts |  |
| Yuki et al, 2019 | * | * | * | * |  | * | * |  | * | * | * | 9 |
| Peterson et al, 2009 | * | * |  | * |  | * | * |  | * | * | * | 8 |
| Savela et al, 2013 |  |  |  | * |  | * | * |  | * | * | * | 6 |
| Niederstrasser et al, 2019 | * | * |  | * |  | * |  |  | * | * | * | 7 |
| Kolehmainen et al, 2020 | * | * |  | Not mentioned |  | * | * |  | * | * | * | 7 |
| Yu et al, 2017 | * | * |  | * |  | * | * |  | * | * | Not mentioned | 7 |
| Gil-Salcedo et al, 2020 |  |  |  | * |  | * | * |  | * | * | * | 6 |
| Borda et al, 2020 | * | * |  | * |  | * | * |  | * | * | * | 8 |
| Abe et al, 2020 | * | * |  | * |  | * | * |  | * | * | * | 8 |
| Pérez-Tasigchana et al, 2020. | * | * |  | Not mentioned |  | * | * |  | * | * |  | 6 |

*: means a star for each study got in the items.

Explanation: 1. The study of Savela et al. conducted the cohort in business men and Gil-Salcedo et al. conducted among British civil servants; the subjects in these studies were not representative of the general population, so they were not allowed to gain the star for the component of representativeness; 2. We assigned a star if the exposure is objective measurement, otherwise a star will not be assigned if the exposure was assessed with questionnaires; 3. In comparability, the most important factors are physical activity, sex, and age, other factors were considered as additional factors; 4. In the study from Pérez-Tasigchana et al., more than 50% of participants cannot be followed up for long-term analyses, which was not allowed to assigned a star; 5. The information that was not mentioned will not be assigned a star.
