# Supplementary material for: Effect of physical activity on the risk of frailty: A systematic review and meta-analysis
Source: PLoS One. 2022 Dec 1;17(12):e0278226. doi: 10.1371/journal.pone.0278226 (PMC9714708; doi:10.1371/journal.pone.0278226)
Supplement: S3 Table — (DOCX) [file pone.0278226.s003.docx]

**Table S3. Grade evidence profile on the association of physical activity with risk of frailty**

| Grade criteria* | Rating | Footnotes | Quality of the evidence |
| --- | --- | --- | --- |
| Study design | Low | Cohort studies | ⊕⊕◯◯ Low |
| Limitations | 0 Point | No serious limitations |  |
| Risk of Bias | 0 Point | No serious |  |
| Inconsistency | - 1 point | A considerable heterogeneity |  |
| Indirectness | 0 Point | No serious |  |
| Imprecision | 0 Point | No serious |  |
| Publication bias | 0 Point | Undetected |  |
| Large effect | 0 point | Pooled effect was 0.59 (95% CI: 0.51-0.67)  More than 2 studies with effect of less than 0.5 |  |
| Plausible confounding | +1 point | No plausible confounding |  |

* Number of studies: 14; Patient or population: Moderate and order age adults; Follow-up: 3-26 years; Exposure: Physical activity; Outcome: frailty risk; Measurements: Measurement with Questionnaire or accelerometry sensor.
