# Supplementary material for: Effect of physical activity on the risk of frailty: A systematic review and meta-analysis
Source: PLoS One. 2022 Dec 1;17(12):e0278226. doi: 10.1371/journal.pone.0278226 (PMC9714708; doi:10.1371/journal.pone.0278226)
Supplement: S1 Fig — (DOCX) [file pone.0278226.s004.docx]

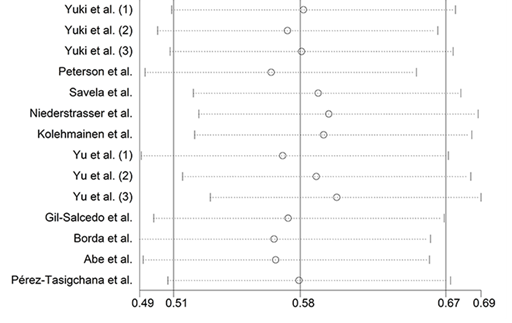


**Figure S1.** Sensitivity analyses on the association between physical activity and the risk of frailty by excluding one study each time.
