# Supplementary material for: Effect of physical activity on the risk of frailty: A systematic review and meta-analysis
Source: PLoS One. 2022 Dec 1;17(12):e0278226. doi: 10.1371/journal.pone.0278226 (PMC9714708; doi:10.1371/journal.pone.0278226)
Supplement: Attachment — Submitted filename: PONE-D-22-03407.docx [file pone.0278226.s007.docx]

PONE-D-22-03407: This manuscript presents a systematic review and meta-analysis of Effect of physical activity on the risk of frailty. The authors conducted a systematic search, identifying 10 relevant studies. Study quality was evaluated using the Newcastle-Ottawa Scale (NOS). Meta-analysis was conducted the random-effects models.

The authors claim there was no systematic review and meta-analysis on the topic. But my search identifies 4 reviews. However, the systematic search demonstrates that there are enough studies to warrant a systematic review and meta-analysis. The quantitative analysis (including sensitivity analyses) appears to have been conducted carefully and appropriately, and the results should be of substantial interest to PLOS ONE readers and the environmental health community.

The manuscript omits several “best practices” that should be part of any systematic review, including preparation of a protocol, a clearly stated study question, and application of preferred approaches for evaluating and reporting risk of bias (or study quality, represented here by the NOS). The authors state that their review was conducted in accordance with the PRISMA guidelines, but several PRISMA recommendations have not been followed. An important challenge for this meta-analysis regardless of the endpoint, is that each study incorporates its own exposure contrast/defintion. The manuscript’s Discussion should address the interpretation of an effect estimate derived from meta-analysis of studies that differ in this important feature. It is also critical for the forest plots and tables to display the exposure contrast for each study. The manuscript contains multiple instances of inconsistent results reporting across the text, tables and figures; some specific instances are reported below.

Grammatical errors are found in places in the text.

**Detailed Comments**

The background should be improved by explaining the mechanism linking physical activity and frailty. There have been several systematic reviews and meta-analyses (e.g., de Vries NM, van et al. 2012;11:136–49, Theou et al 2011, Puts et a; 2017, etc and more recently a protocol by Pan et al 2019). The authors should discuss these reviews and articulate why there is the need for another review. The gap in these previous reviews should be articulated

L3/4: The objective should be stated in accordance with Population Exposure Comparator Outcome statement

L7: This is incorrect. The definition of physical activity varied widely among the studies. In some studies, it was defined as Vigorous/moderate physical activity, or exercise, or hard physical activity

>3 times/week, etc. How can the authors have derived highest and lowest PA.

L29: insert 'a' between 'at' and 'speed'

L32” delete pls ‘the’

L#36 and L#37 are the same-repetition.

L40: pls change 'preventive effect' to 'impact'

In the last sentence of the introduction, the objective of the systematic review was provided. It would be more clearer, and in accordance with recommendations by the PRISMA Statement (BMJ, 2009; 339:b2700), to identify some specific question that the systematic review is intended to address. The questions might include the populations, exposure, comparator, and outcomes of interest. These questions would also support the criteria and decisions made in literature search terms and screening/ selection of studies. The reason for restricting the studies to follow-up design should be clearly articulated in the background.

**Search strategy**

The PRISMA guideline recommends registration of a systematic review. The PRISMA checklist (#8) recommends presentation of the full electronic search strategy for at least one database – not provided in this manuscript.

The search strategy should be structured according to population, exposure comparator, outcome and study design.

The Eligibility criteria did not follow the PECO (S) statement. It should also be structured in accordance with Population, Exposure, Comparator, outcome (s) and study design (PECOS) statement. When stating the inclusion criteria (for example for Population) should also state the reasons for exclusion. Most of what the authors have there as inclusion and exclusion criteria are not; in accordance with the PRISMA's recommendation. For example, how can effect estimate be an inclusion/exclusion criteria. Are the authors not encouraging publication bias. This is a systematic review and a meta-analysis (which also both quantitative and qualitative reporting).

L67: With the PA types specified, the authors should give examples.

L70-71: If the authors had followed the PRISMA recommendations closely. some of what they have here as exclusion criteria would not be here. For example, animal studies, reviews, trials, letters, abstracts, etc would have been removed with search filters. Please, delete this. You did not apply your inclusion/exclusion criteria before excluding them

The authors who did the data extraction and assessment should be identified

L77-80: Newcastle-Ottawa Scale and scoring of studies: I have concerns about the use of an overall scale or score to denote study “quality.” Moreover, the developers of the PRISMA Statement do not recommend this. Some of the evaluation components may have greater impact on determining the value of a particular study for the systematic review. Others, in particular the evaluation of confounding, need to be interpreted for the direction the bias is imposing on the risk estimate (e.g., away from or toward the null). That said, it appears that the NOS scale was used to group studies in sensitivity analyses, rather than to exclude studies from the review. This is an appropriate use of scoring, although I would not know how to interpret the scale of 7 out of 9 in terms of what it really means for “quality.”

The authors should provide detailed explanation of how the NOS was applied. On comparability what are the major and minor confounders considered. A table of NOS assessment including detailed consideration for comparability for each study should be provided in the appendix.

L82: Some of the studies reported OR, others reported RR and some also reported HR. RR, HR are approximated to OR only when OR<10%. The outcome of interest is not a rare outcome and why RR=HR=OR assumption in this study. There are other ways of converting OR to HR or RR and the vice versa.

L87: I realized that the authors reported RR in the tables and ES (effect estimates) in the forest plots. Please, change all RR to ES because the measure of effect were not consistent.

In Table 1 show exposure contrast for PA. This should also be done for the plots.

L100: The flow chart was a bit messy.

For example, 0 articles identified through other sources. What are those other sources? pls delete.

Among the 6424 articles were RCTS you excluded. Are these RCTs on PA and fraility? If yes why delete them because they are also longitudinal designs.

I strongly recommend that the 8 studies that did not provide OR/HR/RR and were excluded should be included in your qualitative analysis.

L107: Provide an exposure contrast for physical activity in Table 1 and this should also be shown in the forest plots.

L130-132: The included studies varied widely on the way in which fraility and PA were measured. For example they reported PA frequency, or PA volum or PA intensity and PA duration. According to the authors PA is a multi-dimensional module and different PA assessments may reflect different facets. I do think the authors need to report overall summary effects. I recommend forest plot should reflect these differences in exposure definition and (summary estimates for each definition) also show PA contrast (e.g., low vs high).

I think the results can be presented descriptively without going through the meta-analysis, subgroup analysis and meta-regresssion

L136-137: The authors should know that funnel visualization and the Beggs method do not perform well with few studies.

L185-187: This is the more reason why i think, pooling the results is not a good idea. Analysis could be done for each PA definition as suggested above. Again gave the fact that the studies are not many.

L23-224: The authors should also discuss inherent limitations in included studies.

Other limitations in the study include the assessment of quality as against the risk of bias.
