# Supplementary material for: Effect of physical activity on the risk of frailty: A systematic review and meta-analysis
Source: PLoS One. 2022 Dec 1;17(12):e0278226. doi: 10.1371/journal.pone.0278226 (PMC9714708; doi:10.1371/journal.pone.0278226)
Supplement: Attachment — Submitted filename: R1_point-to-point response.docx [file pone.0278226.s008.docx]

**Re:** **Effect of physical activity on the risk of frailty:** **a systematic review and meta-analysis (PONE-D-22-03407)**

We thank the reviewers and editors for his/her valuable comments, our point-by-point responses are shown below the comments. The changes made in the revised manuscript were highlighted in red.

**C** = comment; **R** = reply

**#To reviewer 1**

**C1:** The authors claim there was no systematic review and meta-analysis on the topic. But my search identifies 4 reviews. However, the systematic search demonstrates that there are enough studies to warrant a systematic review and meta-analysis. The quantitative analysis (including sensitivity analyses) appears to have been conducted carefully and appropriately, and the results should be of substantial interest to PLOS ONE readers and the environmental health community.

**R1**: Thanks for reviewer’s comment. We appreciate the reviewers for the recognition of our quantitative analysis and the significance of the results. We have carefully updated literature in the manuscript accordingly:

“Physical activity (PA) can regulate the function of multiple systems in the body [1]; the dysfunction of these body system can further contribute to the development of frailty [1]. A systematic review [15], a scoping review [16], and a systematic review and meta-analysis [17] synthesized the evidence and found that exercise training can reduce the level of frailty and improve the prognosis of frailty among older adults. However, epidemiological studies on the relationship between PA and frailty did not reach to a consistent conclusion. For instance, Trombetti [18] and Pérez-Tasigchana [19] did not find any association between PA and frailty, while Borda [20] and Savela [21] reported that PA was a protective factor for frailty. A meta-analysis found that physical exercise therapy could improve mobility and physical functioning in elderly patients suffering from mobility problems, disability and/or multi-morbidity [22]; however, more original studies have been reported in the following decade, making it necessary to update the synthetic evidence. A more recent systematic review including four randomized controlled trials and two prospective cohort studies found that physical activity might be an effective intervention for preventing frailty mong people aged 65 years and older [23]; nevertheless, the intervention measures varied largely among four RCTs and one included RCT adopted combined nutrition and exercise interventions, limiting the application of the results. Moreover, this study[23] only included two cohort studies, but more cohort studies have been reported [18-21,24-30]. In addition, there are many evaluation methods for frailty, and no unified standard has been formed [31]. Whether different methods of frailty assessment influence the association between PA and frailty onset is unclear.” **[lines 34-52]**

**C2:** The manuscript omits several “best practices” that should be part of any systematic review, including preparation of a protocol, a clearly stated study question, and application of preferred approaches for evaluating and reporting risk of bias (or study quality, represented here by the NOS). The authors state that their review was conducted in accordance with the PRISMA guidelines, but several PRISMA recommendations have not been followed. An important challenge for this meta-analysis regardless of the endpoint, is that each study incorporates its own exposure contrast/defintion. The manuscript’s Discussion should address the interpretation of an effect estimate derived from meta-analysis of studies that differ in this important feature. It is also critical for the forest plots and tables to display the exposure contrast for each study. The manuscript contains multiple instances of inconsistent results reporting across the text, tables and figures; some specific instances are reported below.

**R2**: Thanks for valuable comments. The whole manuscript has carefully revised accordingly.

**C3:** Grammatical errors are found in places in the text.

**R3:** Thanks for comments. We have checked the gramma and revised the manuscript.

**C4:** The background should be improved by explaining the mechanism linking physical activity and frailty. There have been several systematic reviews and meta-analyses (e.g., de Vries NM, van et al. 2012;11:136–49, Theou et al 2011, Puts et a; 2017, etc and more recently a protocol by Pan et al 2019). The authors should discuss these reviews and articulate why there is the need for another review. The gap in these previous reviews should be articulated.

**R4:** Thanks for the good suggestion. The background in the introduction has been updated as follows:

**“**Physical activity (PA) can regulate the function of multiple systems in the body [1]; the dysfunction of these body system can further contribute to the development of frailty [1]. A systematic review [15], a scoping review [16], and a systematic review and meta-analysis [17] synthesized the evidence and found that exercise training can reduce the level of frailty and improve the prognosis of frailty among older adults. However, epidemiological studies on the relationship between PA and frailty did not reach to a consistent conclusion. For instance, Trombetti [18] and Pérez-Tasigchana [19] did not find any association between PA and frailty, while Borda [20] and Savela [21] reported that PA was a protective factor for frailty. A meta-analysis found that physical exercise therapy could improve mobility and physical functioning in elderly patients suffering from mobility problems, disability and/or multi-morbidity [22]; however, more original studies have been reported in the following decade, making it necessary to update the synthetic evidence. A more recent systematic review including four randomized controlled trials and two prospective cohort studies found that physical activity might be an effective intervention for preventing frailty mong people aged 65 years and older [23]; nevertheless, the intervention measures varied largely among four RCTs and one included RCT adopted combined nutrition and exercise interventions, limiting the application of the results. Moreover, this study[23] only included two cohort studies, but more cohort studies have been reported [18-21,24-30]. In addition, there are many evaluation methods for frailty, and no unified standard has been formed [31]. Whether different methods of frailty assessment influence the association between PA and frailty onset is unclear.

Pan and colleagues published a registered protocol for systematic review and meta-analysis to demonstrate the association between PA and the risk of frailty in the old community-dwelling residents [32]; however, the related systematic review and meta-analysis has not been reported in the past three years. What’s more, this protocol [32] did not mention how to define frailty and whether synthesize the evidence by frailty definition or not.

Therefore, we conducted this systematic review and meta-analysis by synthesize evidence from cohort studies to examine the effect of PA on the onset of frailty among the community-dwelling middle and older age adults, so to provide evidence for frailty prevention for middle and order age adults.**” [Line 34-61]**

**C5:** L3/4: The objective should be stated in accordance with Population Exposure Comparator Outcome statement

**R5:** Thanks for your comment, we updated the objective in the abstract and in the introduction as follows:

“This systematic review with meta-analysis aimed to evaluate the effect of PA on the onset of frailty in the community-dwelling middle and order age adults by pooling data from cohort studies.” **[Lines 3-5]**

“Therefore, we conducted this systematic review and meta-analysis by synthesize evidence from cohort studies to examine the effect of PA on the onset of frailty among the community-dwelling middle and older age adults, so to provide evidence for frailty prevention for middle and order age adults.” **[Lines 58-61]**

**C6:** L7: This is incorrect. The definition of physical activity varied widely among the studies. In some studies, it was defined as Vigorous/moderate physical activity, or exercise, or hard physical activity>3 times/week, etc. How can the authors have derived highest and lowest PA.

**R6:** Thank you for the comment. The definition of physical activity indeed varied widely in these studies. However, in these studies, the PA indicator often was transformed to categorical variable, and then the effect of PA on the frailty risk was estimated by comparing the high category with low category (such as high vs. Low, vigorous vs. sedentary), or by comparing the low category with high category (such as Sedentary vs. Vigorous). To facilitate the pooled analysis, we transformed the comparison of “low category with high category” to the comparison of “high category with low category” by using the reciprocal method suggested by Cooper and colleague [Ref.36 : Cooper HM, Hedges LV, Valentine JC (2019) The handbook of research synthesis and meta-analysis. New York: Russell Sage Foundation].

Now we have updated the table 1 and figure 2, adding the comparison of PA levels.

**C7:** L29: insert 'a' between 'at' and 'speed'

**R7:** Thanks for your comment, we have inserted “a” accordingly. **[Line 30]**

**C8:** L32” delete pls ‘the’

**R8：**Thanks for your comment, “the” has been deleted accordingly. [**Line 33]**

**C9:** L#36 and L#37 are the same-repetition.

**R9：**Thanks for raising this question, we have revised the paragraph and deleted the repeated content. **[Line35]**

**C10:** L40: pls change 'preventive effect' to 'impact'

**R10**: Thanks for your comment, the paragraph in which the phrase “preventive effect” was placed has been revised.

**C11:** In the last sentence of the introduction, the objective of the systematic review was provided. It would be more clearer, and in accordance with recommendations by the PRISMA Statement (BMJ, 2009; 339:b2700), to identify some specific question that the systematic review is intended to address. The questions might include the populations, exposure, comparator, and outcomes of interest. These questions would also support the criteria and decisions made in literature search terms and screening/ selection of studies. The reason for restricting the studies to follow-up design should be clearly articulated in the background.

**R11:** Thanks for suggestion. We have revised accordingly as follows:

“Physical activity (PA) can regulate the function of multiple systems in the body [1]; the dysfunction of these body system can further contribute to the development of frailty [1]. A systematic review [15], a scoping review [16], and a systematic review and meta-analysis [17] synthesized the evidence and found that exercise training can reduce the level of frailty and improve the prognosis of frailty among older adults. However, epidemiological studies on the relationship between PA and frailty did not reach to a consistent conclusion. For instance, Trombetti [18] and Pérez-Tasigchana [19] did not find any association between PA and frailty, while Borda [20] and Savela [21] reported that PA was a protective factor for frailty. A meta-analysis found that physical exercise therapy could improve mobility and physical functioning in elderly patients suffering from mobility problems, disability and/or multi-morbidity [22]; however, more original studies have been reported in the following decade, making it necessary to update the synthetic evidence. A more recent systematic review including four randomized controlled trials and two prospective cohort studies found that physical activity might be an effective intervention for preventing frailty mong people aged 65 years and older [23]; nevertheless, the intervention measures varied largely among four RCTs and one included RCT adopted combined nutrition and exercise interventions, limiting the application of the results. Moreover, this study [23] only included two cohort studies, but more cohort studies have been reported [18-21,24-30]. In addition, there are many evaluation methods for frailty, and no unified standard has been formed [31]. Whether different methods of frailty assessment influence the association between PA and frailty onset is unclear.

Pan and colleagues published a registered protocol for systematic review and meta-analysis to demonstrate the association between PA and the risk of frailty in the old community-dwelling residents [32]; however, the related systematic review and meta-analysis has not been reported in the past three years. What’s more, this protocol [32] did not mention how to define frailty and whether synthesize the evidence by frailty definition or not.

Therefore, we conducted this systematic review and meta-analysis by synthesize evidence from cohort studies to examine the effect of PA on the onset of frailty among the community-dwelling middle and older age adults, so to provide evidence for frailty prevention for middle and order age adults.**”** **[Line34-61]**

**C12:** The PRISMA guideline recommends registration of a systematic review. The PRISMA checklist (#8) recommends presentation of the full electronic search strategy for at least one database – not provided in this manuscript. The search strategy should be structured according to population, exposure comparator, outcome and study design. The Eligibility criteria did not follow the PECO (S) statement. It should also be structured in accordance with Population, Exposure, Comparator, outcome (s) and study design (PECOS) statement. When stating the inclusion criteria (for example for Population) should also state the reasons for exclusion. Most of what the authors have there as inclusion and exclusion criteria are not; in accordance with the PRISMA's recommendation. For example, how can effect estimate be an inclusion/exclusion criteria. Are the authors not encouraging publication bias. This is a systematic review and a meta-analysis (which also both quantitative and qualitative reporting).

**R12:** Thanks for reviewer’s comments. The PRISMA guideline recommends registration of a systematic review; however, it is not a mandatory component. In fact, many systematic reviews published even in high-prestigious journals (such as JAMA, Lancet, JACC) also did not with a registration.

As we stated in the manuscript, we searched literatures from three datasets (PubMed, Embase, and Web of science). We used the keywords and retrieval strategy as follows: “(‘physical activity’ OR ‘exercise’ OR ‘acute exercise’ OR ‘isometric exercises’ OR ‘aerobic exercise’ OR ‘physical exercise’ OR ‘endurance exercise’ OR ‘resistance exercise’ OR ‘strength exercises’ OR ‘training’ OR ‘exercise training’, OR ‘combined training’, OR ‘weight-lifting’ OR ‘running’ OR ‘jogging’ OR ‘swimming’ OR ‘walking’ OR ‘yoga’ OR ‘Tai chi’ OR ‘daily activity’ OR ‘lifestyle’ OR ‘sport’) AND (‘frailty’ OR ‘frailness’ OR ‘frailty syndrome’ OR and ‘debility’)”. We displayed the detailed search strategy in the supplementary Table S1. We also did a reverse reference citation tracking to find potential reports. The flow-chart of literature selection can be seen in Figure 1. **[Lines 64-75, supplementary table S1]**

We sincerely pay more attention to the publication bias. The misunderstanding might due to the incorrect description. To make clear for readers, we have revised the paragraph related the inclusion and exclusion criteria accordingly: “Inclusion criteria were as follows: the exposure of PA including exposure intensity, frequency, duration, volume, step, or any specific type (such as leisure-time physical activities, occupational activities, and exercise, etc.) was reported; frailty and its assessment approaches (physical frailty, multidimensional approach, accumulation of disability, etc.) were reported ; design of cohort studies; middle and order age healthy adults; the relationship between PA and frailty was evaluated; the study was published in English. Cross-sectional studies, animal studies, trials, reviews, editorials, letters, abstracts, and studies lacking data to manifest the relationship between PA and frailty were excluded after reviewing title, abstract or full-text.” **[Lines 76-83]**

**C13:** L67: With the PA types specified, the authors should give examples.

**R13:** Thanks for suggestion. The examples of specific PA types were given as follows: “(such as leisure-time physical activities, occupational activities, and exercise, etc.)” **[Line: 77-78]**

**C14**: L70-71: If the authors had followed the PRISMA recommendations closely. some of what they have here as exclusion criteria would not be here. For example, animal studies, reviews, trials, letters, abstracts, etc would have been removed with search filters. Please, delete this. You did not apply your inclusion/exclusion criteria before excluding them

**R14:** Thanks for your comments. W have updated the inclusion and exclusion criteria as follows: **“**Inclusion criteria were as follows: the exposure of PA including exposure intensity, frequency, duration, volume, step, or any specific type (such as leisure-time physical activities, occupational activities, and exercise, etc.) was reported; frailty and its assessment approaches (physical frailty, multidimensional approach, accumulation of disability, etc.) were reported ; design of cohort studies; middle and order age healthy adults; the relationship between PA and frailty was evaluated; the study was published in English. Cross-sectional studies, animal studies, trials, reviews, editorials, letters, abstracts, and studies lacking data to manifest the relationship between PA and frailty were excluded after reviewing title, abstract or full-text**” [Lines: 76-83]**

**C15:** The authors who did the data extraction and assessment should be identified

**R15:** Thanks for your comments, we have added such information in the section of authors’ contributions: “Data extraction and assessment: PH, WJZ & WDS”. **[Lines: 284]**

**C16:** L77-80: Newcastle-Ottawa Scale and scoring of studies: I have concerns about the use of an overall scale or score to denote study “quality.” Moreover, the developers of the PRISMA Statement do not recommend this. Some of the evaluation components may have greater impact on determining the value of a particular study for the systematic review. Others, in particular the evaluation of confounding, need to be interpreted for the direction the bias is imposing on the risk estimate (e.g., away from or toward the null). That said, it appears that the NOS scale was used to group studies in sensitivity analyses, rather than to exclude studies from the review. This is an appropriate use of scoring, although I would not know how to interpret the scale of 7 out of 9 in terms of what it really means for “quality.” The authors should provide detailed explanation of how the NOS was applied. On comparability what are the major and minor confounders considered. A table of NOS assessment including detailed consideration for comparability for each study should be provided in the appendix.

**R16**: Thanks for your comments. In our manuscript, we provided the quality assessment results by using NOS (supplementary table S2). Now, we updated it and provided a more detailed table. Also, the detailed explanation was provided. **[see supplementary table S2]**

**C17:** L82: Some of the studies reported OR, others reported RR and some also reported HR. RR, HR are approximated to OR only when OR<10%. The outcome of interest is not a rare outcome and why RR=HR=OR assumption in this study. There are other ways of converting OR to HR or RR and the vice versa.

**R17:** Thanks for your comments. Considering that only 2 records reported HR, and other 12 records reported OR, we pooled the results using effect estimates (ES) across the manuscript. Moreover, we further conducted subgroup analysis by effect estimates, and similar protective effect was observed for the studies using OR (pool ES: 0.60, 95% CI: 0.52 - 0.70) and the studies using HR (pool ES: 0.54, 95% CI: 0.38 - 0.77). We also updated all the results across the manuscript. **[see Table 2]**

**C18:** L87: I realized that the authors reported RR in the tables and ES (effect estimates) in the forest plots. Please, change all RR to ES because the measure of effect were not consistent.

**R18**: Thanks for comments, we changed the RR with ES across the manuscript.

C19: In Table 1 show exposure contrast for PA. This should also be done for the plots.

**R19**: Thanks for comments, we added the exposure contrast for PA in the forest plots. **[see table S2, Figure 2]**

**C20:** L100: The flow chart was a bit messy. For example, 0 articles identified through other sources. What are those other sources? pls delete.

**R20**: Thanks for your comments. We updated the flow-chart accordingly. **[see Figure 1]**

**C21:** Among the 6424 articles were RCTS you excluded. Are these RCTs on PA and frailty? If yes why delete them because they are also longitudinal designs.

**R21:** Thanks for your comments. Among the RCTs we excluded, most of the RCTs were not relevant to the topic of this study. Two RCTs (de Souto Barreto et al., 2018; Li et al., 2010) conducted with multidomain intervention with frailty; however, PA is a subitem of the intervention and the effects of PA were not reported or can be calculated. Nagai et al (Nagai et al., 2018) conducted a RCT to explore PA combined with resistance training on frailty symptoms; however, the study just reported the difference between groups and cannot estimate the effect size of each single component. Given the limited available evidence from RCT study and the target to ascertain the effect of PA on frailty incidence, we limited the included study to prospective cohort study to prevent the potential bias from different study designs. In fact, in the inclusion criteria of this study, we also limited the included study to cohort study.

**C22:** I strongly recommend that the 8 studies that did not provide OR/HR/RR and were excluded should be included in your qualitative analysis.

**R22:** Thanks for your comments. Among 8 studies we excluded, 5 studies (Cheung et al., 2020; Ye et al., 2020; Lorenzo-López et al., 2019; Pao et al., 2018; Rogers et al., 2017) focused on the changes in frailty or the trajectories of frailty, which did not report the association between PA and frailty. One study from US explored the relationship between physical activity and frailty among older adults based on hourly accelerometry data, and the study showed that each frailty point corresponded a 7% lower mean hourly activity counts per minute by using mixed effects linear regression model (Huisingh-Scheetz et al., 2017). One study from Spain used multivariate linear regression models and showed that compared with participants with a continued regular PA frequency, participants with a decreased frequency were significantly more overall frailty (β = 1.31; 95% confidence interval = 0.99-1.63) (Zhang et al., 2020). One study conducted among 604 Japanese women showed that high exercise and high dietary varieties group was associated with lower risk of frailty (OR, 0.38, 95% CI, 0.15-0.92); however, this study did not report the effect between PA and frailty solely (OSUKA et al., 2019).

Given to the limited evidence on PA and frailty left, we added the following sentences in the discussion section: “In addition, similar protective effect of PA on the frailty was also reported by two cohort studies using linear regression model [40,41]. Based on hourly accelerometry data, Huisingh-Scheetz et al. found that each frailty point corresponded a 7% lower mean hourly activity counts per minute among older adults by using mixed effects linear regression model [40]. Zhang et al. used multivariate linear regression models and showed that compared with participants with a continued regular PA frequency, participants with a decreased frequency were significantly more overall frailty [41].” **[Line 197-203, see figure 1]**

**C23:** L107: Provide an exposure contrast for physical activity in Table 1 and this should also be shown in the forest plots.

**R23:** Thanks for comments, we added such information in **Table 1, Figure 2, and supplementary Figure S2**.

**C24:** L130-132: The included studies varied widely on the way in which frailty and PA were measured. For example, they reported PA frequency, PA volume or PA intensity and PA duration. According to the authors PA is a multi-dimensional module and different PA assessments may reflect different facets. I do think the authors need to report overall summary effects. I recommend forest plot should reflect these differences in exposure definition and (summary estimates for each definition) also show PA contrast (e.g., low vs high).

**R24:** Thanks for comments. We have provided the pooled results from all included studies and each PA indicators in table 2. Now we also added the forest plots by PA indicators in supplementary materials (see Supplementary Figure S2) and added the following sentence to the results section: **“**The forest plot of association between PA and frailty risk by PA indicators was shown in Figure S2.**” [Lines 179-180]**

**C25:** I think the results can be presented descriptively without going through the meta-analysis, subgroup analysis and meta-regression

**R25:** Thanks for the comments. In fact, almost the generalizability of the included studies was limited in specific regions. Hence, from the perspective of public health and guiding practice, we remain our view that it is necessary to conduct a pooled analysis and provide the synthetic results. Therefore, we conducted meta-analysis and subgroup analysis, drew forest plot of the relationship between PA and fragility risk and forest plot grouped according to PA indicators. We also did the meta-regression was conducted to assess the potential sources of heterogeneity.

**C26:** L136-137: The authors should know that funnel visualization and the Begger’s method do not perform well with few studies.

**R26**: Thanks for comments. Considering that only 14 records from ten studies were included, and the regression method is more sensitive than the rank correlation approach (Sterne et al., 2001), we conducted funnel plot together with Begger’s test, and Egger’s test to test the publication bias. The p-values from Begger’s test and Egger’s test were shown in **Table 2**.

**C27:** L185-187: This is the more reason why i think, pooling the results is not a good idea. Analysis could be done for each PA definition as suggested above. Again gave the fact that the studies are not many.

**R27:** Thanks for comments. In fact, we also did the analysis by each PA indicator, we added the results to table 2 and the forest plot to supplementary materials. **[See Table 2 and Supplementary Figure S2].**

**C28:** L23-224: The authors should also discuss inherent limitations in included studies. Other limitations in the study include the assessment of quality as against the risk of bias.

**R28:** Thanks for comments. The discussion has been updated by including the discussion of the limitations in included studies.

**“**Nevertheless, some potential limitations should be recognized in our studies. First, the frailty in the included studies was defined by different approaches, and PA was assessed by using different scales, these may result in information bias and thus may affect the consistency of the pooled results; however, the sensitivity and stratified analysis did not find any significant change. Then, the heterogeneity in some pooled analyses was significant, this may be due to the diversity of the characteristics of the subjects, the measurement of exposure, and the measurement of outcome in each study. For instance, the study conducted by Savela et al. was limited in Caucasian men of high socioeconomic status [21]; most of the included studies used self-report questionnaire to derive levels of PA, but the survey questions used in these studies varied largely. Hence, more studies with the same PA measurements and same frailty evaluation method are needed to further validate our results. Third, almost all the included studies assessed PA at the baseline, however, the longitudinally change of PA level and whether these changes influenced the incidence of frailty were unclear. This should be studied in the future. Fourth, because of the long follow-up in some studies, the missing data from those who died during follow-up may dilute the association between PA and frailty; therefore, more effective analyzed methods such as competing risk model should be considered.” **[Lines 262-276]**

**#To reviewer 2**

**C1:** Introduction - Page 3 line 37: Please remove the duplicate sentence: "such as the musculoskeletal, cardiorespiratory, endocrine, and nervous systems"

**R1:** Thanks for your suggestion, we have revised the paragraph. **[Lines 34-52]**

C2：Methods - Page 4 line 66: I have the impression the inclusion criteria could be improved and more detailed. Could you please use the PICO format to assist readers understanding the type of study included? I believe it is unclear how frailty outcomes were considered in this review. Also, there is no information about the population (please add the age range, if applicable). Apologies if I missed it.

**R2:** Thanks for your suggestion. We added the age range to the fourth column in table 1. We also updated the description of inclusion and exclusion criteria accordingly.

**“**Inclusion criteria were as follows: the exposure of PA including exposure intensity, frequency, duration, volume, step, or any specific type (such as leisure-time physical activities, occupational activities, and exercise, etc.) was reported; frailty and its assessment approaches (physical frailty, multidimensional approach, accumulation of disability, etc.) were reported ; design of cohort studies; middle and order age healthy adults; the relationship between PA and frailty was evaluated; the study was published in English. Cross-sectional studies, animal studies, trials, reviews, editorials, letters, abstracts, and studies lacking data to manifest the relationship between PA and frailty were excluded after reviewing title, abstract or full-text.**” [Lines 76-83]**

C3: Table 2. The title suggests subgroup analyses only, but I think you also included the overall analyses. Please review this to inform readers table 2 includes overall and subgroup meta-analyses. Also, it is difficult to quickly identify significant poolings. Could you please bold the significant results?

**R3**: Thanks for the good suggestion. We revised the title of Table 2 and we also bolded the significant results in table 2.

**C4:** Confounders. Please add information about confounders. I noticed you added a sentence in your discussion, but this may not be enough.

**R4:** Thanks for the suggestion. In fact, we have showed the confounders of each included study in the general characteristics table **[Table 1]**.

**C5:** Adjusted analysis. Please add information on whether you included unadjusted and adjusted results in the meta-analysis. I have the impression this is unclear in your manuscript. Moreover, could you conduct meta-analysis including only adjusted analysis?

**R5:** Thanks for comments. Considering that the results of the included studies would be more reliable after adjusting for potential confounders than the unadjusted results, in this meta-analysis, we evaluated the pooled effects based on the adjusted results in each included study.

We added such information in section of statistical analysis: **“**A meta-analysis was conducted to estimate the overall pooled effect estimates (ES) based on the adjusted odds ratio or hazard ratio and 95% confidence interval (CI), by comparing the highest with the lowest levels of PA.” **[Line 105-107]**

**C6:** Page 7 line 133. I find it interesting that you described moderate heterogeneity for your main meta-analysis. I believe this is more likely to be classified as substantial heterogeneity (following the Cochrane Handbook). Please justify why a heterogeneity of 70% should be considered moderate in your review or reclassify it.

**R6:** Thanks for comments. We rechecked the classification of heterogeneity according to the Cochrane Handbook, and found that described a heterogeneity of 70% as moderate heterogeneity was inaccurate. We correct it according to your suggestions.

**“**The random-effects model was used to calculate the pooled effect as the substantial heterogeneity was found (I*^2^* = 70.0%, *P*_-heterogeneity_ *<* 0.001).**” [Line 162-163]**

**C7:** Suggestion: To strengthen your review, could you include the GRADE approach to summarise the certainty of evidence?

**R7:** Thanks for your suggestions. We further included the GRADE approach to evaluate the quality of the evidence. We found that the quality of the evidence from the outcomes evaluated by the GRADE system was classified as moderate (showed in Supplementary Table S3), and substantial heterogeneity was the main reason responsible for the limited quality of the evidence.

We accordingly added the following sentence to the section of Methods: **“**The Grading of Recommendations Assessment, Development and Evaluation (GRADE) approach was used to evaluated the quality of the body of evidence [29]. The GRADE approach was based on considerations such as study design, risk of bias, inconsistency, imprecision, indirectness, publication bias and other aspects reported by the included studies. The quality of the evidence was characterized as high, moderate, low, or very low.**” [Line 93-98]**

We accordingly added the following sentence to the section of Results: **“**The quality of the evidence from the outcomes evaluated by the GRADE system was assessed as moderate (Supplementary Table S3). Substantial heterogeneity was the main reason responsible for the limited quality of the evidence.**”** **[Line 132-134]**
