# Supplementary material for: Effect of physical activity on the risk of frailty: A systematic review and meta-analysis
Source: PLoS One. 2022 Dec 1;17(12):e0278226. doi: 10.1371/journal.pone.0278226 (PMC9714708; doi:10.1371/journal.pone.0278226)
Supplement: Attachment — Submitted filename: R2_point-to-point response.docx [file pone.0278226.s009.docx]

**Re:** **Effect of physical activity on the risk of frailty:** **a systematic review and meta-analysis (PONE-D-22-03407)**

We thank the reviewers and editors for his/her valuable comments, our point-by-point responses are shown below the comments. The changes made in the revised manuscript were highlighted in red.

**C** = comment; **R** = reply

**#To reviewer 1**

**C1:**  The manuscript is technically sound, statistically analysis was carefully conducted, nut i have not seen evidence that the authors have shared their data. The standard of English is improved.

**R1**: Thanks for the reviewer’s comment. This study is a systematic review and meta-analysis; the data was extracted from published research. The data is available by contacting the corresponding author or can be extracted from original published research.

**#To reviewer 2**

**C1:** General comments: Thank you for addressing the reviewers’ comments in the updated version of the manuscript. Your study is interesting, and I believe it is important considering the impact of frailty on public health. I still find this study challenging to understand as the text is too long with important messages difficult to identify (e.g. certainty of evidence). Proofreading could assist, with focus on results and discussion.

**R1**: Thanks for reviewer’s comment. The whole manuscript has carefully revised accordingly.

**C2:** Some specific comments: PRISMA checklist – Registration and protocol - Some of the items you classified as “NA - Not applicable”. This may not be correct as these items are applicable. However, I have the impression you failed to complete them. Please review this and be clear and say “not performed” for items 24 a,b,c if not performed by your team. Or, please explain why they are not applicable.

**R2**: Thanks for your suggestion, we have changed the “NA” with “not performed” in the PRISMA checklist.

**C3:** GRADE - I noticed you include GRADE as recommended – thank you. Yet, I missed seeing in your method/appendix a clear description of the GRADE parameters you used. Also, I believe the certainty of the evidence was not properly incorporated into your discussion, conclusion and abstract. Please review how GRADE is described, applied and reported across the manuscript to assist others in understanding the certainty of your findings.

**R3:** Thanks for your useful comment. We added detailed footnotes of the description of the GRADE approach for Table S3. Furthermore, we added the description of the certainty of the evidence across the manuscript accordingly.

In the abstract, we added the following sentences:

(1) “The quality of evidence was evaluated by using the Grading of Recommendations Assessment, Development and Evaluation (GRADE) approach.” [lines 9-11]

(2) “and the GRADE approach classified the quality of evidence as moderate.” [line 12-13]

(3) “This study with moderate-certainty evidence shows that…” [line 21]

In the conclusion, we added the following sentences:

(1) “Based on available evidence with moderate quality involving 34,943 participants, results from this systematic review and meta-analysis suggest that a higher level of PA was significantly associated with a lower risk of frailty.” [186-188]

(2) “In conclusion, this systematic review and meta-analysis with moderate-certainty evidence suggests that...” [line 274]

**C4:** Methods: Page 6, line 98: Ethical statement is likely irrelevant for this study and could be deleted.

**R4**: Thanks for your useful comment. We have deleted ethical statement in the manuscript.

**C5:** Page 6, line 104: Please include a reference to assist readers in understanding how you are expressing your results. This is the first time I have come across “overall pooled effect estimates (ES)”. combining OR with HR. I have the impression this is not commonly issued nor recommended in the COCHRANE handbook. I could be wrong. Further details on your method may be required to guide readers.

**R5**: Thanks for your important comment. We have included a reference to assist readers in understanding the methods [Ref. 36]. In fact, A Jatho et al. also conducted a meta-analysis by pooling the effect estimates using the adjusted OR, RR or HR and its 95% CI from each included study. Meanwhile, we have further conducted subgroup analysis by effect estimates (odds ratio, hazard ratio) to reveal the effect of the studies reporting OR and HR as a result, respectively.

**C6:** Page 6, line 110. Please write in full “RR” as this is a new abbreviation

**R6**: Thanks for your comment. We have changed “RR” with “ES” in the manuscript.

**C7:** Results: I am concerned you may have included in your Meta-Analysis the same participants more than once (e.g. one participant may have been included in two analyses for different outcomes). For example, Yuki (2) and Yuki (3). Are these participants from the same Taiwan urban cohort with data collected in 2003? Are they the same participants? Please review this to make sure you do not include the same sample more than once in your pooling. Same for the Finish cohort.

**R7:** Thanks for your comments. In fact, Yu et al. conducted the study from different areas: Yu (1) from Hong Kong, Yu (2) from Taiwan urban, and Yu (3) from Taiwan rural; hence, they are not the same population. The study from Yuk displayed the results from different physical activity indicators, we pooled the results and further conducted the subgroup analysis by physical activity indicators.

**C8:** Also, the comparison information in Figure S2 is challenging to understand. For example, you used the same name for comparison groups in different subgroups. You included “active vs inactive” for PA duration with no reference to duration. This seems unclear to me. Could you please rename them or provide further details in the figure.

**R8:** Thanks for your comments. In fact, the names of the comparison information were from the statement of the included studies. To help readers understand, we have renamed the comparison information concretely in Figure S2 and in Figure 2 accordingly.

**C9:** Discussion/Conclusion: Please add a sentence about the certainty of evidence as this should be part of your main findings.

**R9:** Thanks for your suggestion. We added the description of the certainty of the evidence across the manuscript accordingly.

We accordingly added the following sentence to the section of Discussion: “Based on available evidence with moderate quality involving 34,943 participants, results from this systematic review and meta-analysis suggest that a higher level of PA was significantly associated with a lower risk of frailty.” [Line 191-192]

“In conclusion, this systematic review and meta-analysis with moderate-certainty evidence suggests that….” [Line 279-282]
