# Supplementary material for: Effect of physical activity on the risk of frailty: A systematic review and meta-analysis
Source: PLoS One. 2022 Dec 1;17(12):e0278226. doi: 10.1371/journal.pone.0278226 (PMC9714708; doi:10.1371/journal.pone.0278226)
Supplement: Attachment — Submitted filename: R3-comment.docx [file pone.0278226.s010.docx]

**Re:** **Effect of physical activity on the risk of frailty:** **a systematic review and meta-analysis**

We thank the reviewers and editors for his/her valuable comments, our point-by-point responses are shown below the comments. The changes made in the revised manuscript were highlighted in red.

**C** = comment; **R** = reply

**# To Reviewer 2:**

GRADE approach for Table S3:

C1: Please include all parameters with the description you used for GRADE (e.g. Phase of investigation, Study limitations, Inconsistency, Indirectness, Imprecision, Publication bias, Effect size).

R1: Thanks for your comment. We have included all the parameters that the GRADE handbook pointed out and added the footnotes for Table S3.

C2: Effect size: Could you please explain why you upgraded the evidence by 1x point due to the large effect? “2 Large effect: More than 2 studies showed the effect estimates<0.5 after adjusting for confounders. (marked 1 point)”. This parameter often considers the effect size of the pooling you are evaluating, not the estimates from individual studies. In this case, I believe the ES for the pooling is 0.59, which is above 0.5, suggesting no upgrade. Please review it.

R2: Thanks for your important comment. In fact, we have upgraded the evidence by 1 point according the GRADE handbook, which pointed out that the effect <0.5 based on direct evidence could be considered as large effect.

C3: Confounder: Is this related to imprecision? Please clarify this GRADE parameter as this is not clear to the readers. “3 Plausible confounding would change the effect: Reduced effect for ES<<1. (marked 2 points)”

R3: Thanks for your important comment. In fact, this point was pointed out according to Factors that can increase the quality of the evidence in the GRADE handbook. Plausible confounding that would reduce effect for ES<<1 could upgrade by one level. We updated the Table S3 and furtherly updated the footnotes.

C4：Combining OR/HR/RR: I believe OR/HR/RR should not be combined in one metanalysis without appropriate conversations. As this method has been selected for the main analysis, I recommended the main analysis be checked by an experienced statistician before publication. My impression is that the authors do not present enough evidence to support this choice/method. Combining OR/HR/RR is not the standard approach for metanalysis, and the evidence presented by the authors is insufficient (in my view). The reference Jatho et al used another reference that explains how random-effect metanalysis is performed. This paper used an example combining only ORs. In sum, I am concerned with the method used but less concerned with the estimate presented. The combined estimates (OR/HR) are likely to be similar to the OR and HR estimates shown separately. Your review has several included papers reporting OR and HR. I don’t think it is necessary to present an unconventional metanalysis combining them because you have separate metanalysis for OR and HR showing similar estimates.

R4: Thanks for your helpful comment. Indeed, there is limited evidence combined OR/HR/RR in a mate-analysis. We have consulted experienced statisticians at Sun Yat-sen University. Considering that our study included 12 records using OR as effect estimates and only 2 records using HR as effect estimates, we pooled the effect estimates using the adjusted OR/HR and its 95% CI from each included study as Jatho et al did in the main analysis. In the meanwhile, to differ the effect of the studies reporting OR and HR, we further conducted subgroup analysis by effect estimates. For the results of subgroup analysis did not changed remarkably, we still used the results of the main analysis with relatively more included studies.
