# Supplementary material for: Effect of physical activity on the risk of frailty: A systematic review and meta-analysis
Source: PLoS One. 2022 Dec 1;17(12):e0278226. doi: 10.1371/journal.pone.0278226 (PMC9714708; doi:10.1371/journal.pone.0278226)
Supplement: Attachment — Submitted filename: R4_comment-response.docx [file pone.0278226.s011.docx]

**Re: Effect of physical activity on the risk of frailty: a systematic review and meta-analysis (PONE-D-22-03407R3)**

C = Comment， R = Reply

**To Reviewer #2:**

**C1**: GRADE description: Page 6, line 97 – I still find it difficult to understand your GRADE findings. In the method, you mentioned that GRADE was used but did not provide a description of each parameter. Again, in table S3, you mention the GRADE decisions with no descriptions of the parameters (e.g. Study limitations: No serious risk of bias was found. Indirectness: no. Imprecision: no.). Based on the current information provided in the manuscript, it is not possible for readers to understand how you applied GRADE in your study. This needs to be corrected before publication. Please provide a clear description for each GRADE parameter in your method or Table S3 (e.g. Imprecision: We downgraded if there were <400 participants).

**R1**: Thanks for reviewer’s suggestion. We have modified the Table S3, so the information can be clearer displayed.

We also added the following description in the results section: “By using GRADE approach, it is found that substantial heterogeneity was the main reason responsible for the limited quality of the evidence, whereas all plausible confounding factors were considered (Supplementary Table S3). Hence, the quality of the evidence from the outcomes evaluated by the GRADE system was assessed as low as a whole.” [Lines: 134-138]

**C2**: Effect size upgrade (related to what I described above). It is still unclear if you have upgraded (or not) due to the large magnitude of the effect. Could you please review this? I have the impression you should not upgrade this parameter. Your estimate of effect is 0.59 (0.51 to 0.67), which is above 0.5 (the suggested parameter to upgrade is <0.5). Therefore, it should not be upgraded based on effect size. Please note that none of your estimates presents in Table 2 is below 0.5.

**R2**. Thanks for reviewer’s suggestion. We have modified the Table S3, so the information can be clearer displayed. We also added the following description in the results section: “By using GRADE approach, it is found that substantial heterogeneity was the main reason responsible for the limited quality of the evidence, whereas all plausible confounding factors were considered (Supplementary Table S3). Hence, the quality of the evidence from the outcomes evaluated by the GRADE system was assessed as low as a whole.” [Lines: 134-138]

**C3**: Combining OR/HR. There is no quality evidence supporting your method to combine OR/HR in your main metanalysis. You also mentioned that the evidence supporting this approach is limited. If there is no high-quality evidence supporting this decision, please only present the meta-analysis for OR and HR separately. Please note the main findings of your review is based on a questionable Meta-Analysis, and, in my view, this is concerning. I am happy to accept this method if the Editor supports your decision.

**R3**: Thanks for reviewer’s comments. In our study we only included cohort studies, though two methods of logistic regression and Cox regression were used among these studies. We calculated the whole effects by synthesizing results from all included studies (ES=0.59, 95% CI= 0.51-0.67), and did the stratified analysis according their analyzed methods (for logistic regression: ES=0.60, 95% CI= 0.52-0.70); for cox regression, ES =0.54, 95% CI= 0.38-0.77). We believe this can enhance the robustness and credibility of the results.

**C4**: Abstract findings: Most of your results are ORs and the interpretation should be framed in terms of odds, not in terms of risk. ORs help to understand the magnitude of an effect but are often mistaken for relative risk ratios. Please review your abstract to express your results as odds. Some references: Norton EC, Dowd BE, Maciejewski ML. Odds Ratios—Current Best Practice and Use. JAMA. 2018;320(1):84–85. doi:10.1001/jama.2018.6971. Andrade C. Understanding relative risk, odds ratio, and related terms: as simple as it can get. J Clin Psychiatry. 2015 Jul;76(7):e857-61. doi: 10.4088/JCP.15f10150. PMID: 26231012. Once again, I hope some of these comments/suggestions are helpful.

**R4**. Thanks for reviewer’s helpful suggestion. We have modified the abstract accordingly. The “risk” was replaced with the “odds”. We also update the description in the sections of results and discussion.

**To Reviewer #3:**

**C1**: The research procedure of this paper is standardized and the process is comprehensive, which is a complete meta-analysis relatively. However, I note that the authors searched only three databases during the literature search, including PubMed, Embase, and Web of science, omitting The Cochrane Library database. In addition, several important Chinese databases have been omitted, such as CNKI, WanFang Data and the VIP database, and the included studies included samples from Hong Kong and Taiwan, China. How did the authors ensure the scientific validity of the findings?

**R1**: Thanks for reviewer’s comments. We only considered PubMed, Embase, and Web of science, this was because that almost the vast majority of high-quality Chinese Journals are included into these three datasets. The Cochrane Library database was also considered, as PubMed also included the Cochrane Database of Systematic Reviews. Hence, searched three databases of PubMed, Embase, and Web of science could include nearly all publications. In addition, a reverse reference citation tracking was also carried out to search for the possible literature.
